# Engine Brush

**Published:** 1885-02

**Authors:** 


Engine Brush.—The following method presents advantages: Take your engine porte-polisher and insert in it all the bristles it will hold, then drive into the centre of the brush a hard, wood wedge, which should be notched to break off even with the end of the porte. Cut the bristles, leaving them about a quarter of an inch in length. A few minutes previous to using the brush, place it in water to tighten the wedge. This brush is very useful in cleaning the teeth and finishing fillings.

